# Beyond the beats: a systematic review of the underlying inflammatory pathways between atrial fibrillation and cognitive decline

**DOI:** 10.1007/s10072-025-08040-x

**Published:** 2025-02-20

**Authors:** Ana Mónica Machado, Ana Sofia Ferraz, M. Graça Pereira, Fernanda Leite

**Affiliations:** 1 Research Centre in Psychology (Cipsi), School of Psychology, University of Minho, Braga, Portugal; 2 Department of Transfusion Medicine, Santo António University Hospital Center, Porto, Portugal; 3 Public Health and Forensic Sciences, and Medical Education Department, Faculty of Medicine University of Porto, Porto, Portugal; 4 i3S-Institute for Research and Innovation in Health, Porto, Portugal

**Keywords:** Atrial fibrillation, Cognitive decline, Inflammation, Pathophysiology, Therapeutic targets

## Abstract

Atrial fibrillation (AF) and cognitive decline represent significant health challenges with increasing prevalence and significant socioeconomic implications. Emerging evidence suggests a potential link between AF and cognitive decline, including dementia and Alzheimer’s disease, although the underlying mechanisms remain incompletely understood. Inflammation has emerged as a key mediator in cardiovascular and neurological diseases, encouraging an investigation into its role in the atrial fibrillation-cognition association. A systematic search of PubMed, Web of Science, and PsycInfo was conducted to identify relevant studies investigating possible inflammatory mechanisms bridging AF and cognitive decline. Studies were assessed for quality and relevance, and data were synthesized using a narrative approach. Five papers were included, with only two longitudinal studies. Inflammatory biomarkers emerged as significant factors associated with both AF and cognitive decline. Three studies revealed a correlation between high-sensitivity CRP (HS-CRP) levels and cognitive decline in patients with AF, AF patients with cerebral infarction, and elderly individuals with AF. However, conflicting results were observed, as one study did not identify any associations between cognitive decline and HS-CRP levels. The scientific literature on this topic is scarce, and the results of existing studies often lack consistency in their findings, highlighting the need for further research to better understand and prevent this significant health burden in patients with AF. So, the results of this study are expected to inform future research directions and cognitive decline risk stratification, guiding the development of targeted interventions aimed at preserving cognitive function and improving outcomes in patients with AF.

**Supplementary Information:**

The online version contains supplementary material available at 10.1007/s10072-025-08040-x.

## Introduction

The global epidemic of atrial fibrillation (AF) represents a major public health issue and an economic burden for healthcare systems [1]. According to the Global Burden of Disease project more than 46 million individuals with AF were estimated to exist [2] and almost one-third are asymptomatic [3].

The demographic phenomenon of aging worldwide and the increased survival with chronic inflammatory diseases favor the exponential rise of the incidence and prevalence of AF and its comorbidities [2, 4]. Growing scientific evidence associates AF to an increased risk of cognitive decline including dementia and Alzheimer’s disease (AD) [4–16]. Indeed, beyond constituting a major risk factor for stroke and cardiovascular diseases [17], AF is associated with cognitive impairment and dementia, independently of clinically overt previous stroke [4–8, 10, 12–16, 18]. Dementia is the leading cause of disability and dependence [19] and it is expected to reach more than 75 million people worldwide by 2030 and almost double this number by 2050. Although the link between AF and cognitive impairment is established, an important knowledge gap exists on whether this relationship is causal and what pathophysiological mechanisms are inherent.

The association between AF and cognitive decline carries profound clinical significance, presenting challenges in both the diagnosis and management [20, 21]. Notwithstanding research efforts, preventing AF and its comorbidities remains an important challenge for healthcare systems. Therefore, AF, cognitive decline, and/or dementia represent two major public health challenges that are becoming more prevalent [4, 14, 19] with implications on quality of life (QoL), treatment adherence, prognosis, and healthcare resource utilization [4, 11, 14] representing a burden in healthcare systems worldwide [4, 14, 19].

Since 1997, multiple observational studies have explored the relationship between AF and cognitive decline. However, most are small cross-sectional studies with highly selected populations with short follow-ups [22, 23]. Moreover, AF and dementia share several risk factors [24, 25], such as advanced age, diabetes, chronic kidney disease, sleep apnea, hypertension, heart failure, excessive alcohol consumption, and coronary heart disease, that should be considered in the studies investigating the potential causal link between AF and cognitive decline and/or dementia [4, 14, 26]. While AF and cognitive decline have common risk factors, this association appears to exist independently of these factors [4, 14, 26]. However, conclusive evidence regarding a direct causal relationship remains unattainable (4). Several pathophysiological mechanisms have been proposed to explain this association [4, 24] and, in recent years, several research studies have indicated that inflammation plays a significant role in the pathophysiological development of AF, and conversely, AF exacerbates the inflammatory response ( [27, 28]. Moreover, there is evidence associating heightened inflammatory markers with cognitive decline and dementia [29, 30]. The nature of this association and the underlying mechanisms are critical for optimizing patient care and developing targeted interventions. While previous studies have identified associations between AF and cognitive impairment [4, 6, 25, 31] the underlying pathophysiological pathways remain incompletely understood [4]. Given the growing recognition of inflammation as a key mediator in both cardiovascular and neurological diseases [32–34] exploring its involvement in the AF-cognition link represents a critical step toward elucidating the underlying pathophysiology and identifying novel therapeutic targets [4].

The objective of this study was to systematically evaluate and analyze existing literature to assess the association between inflammatory processes in AF and cognitive decline. Specifically, we aim to investigate the presence and strength of evidence supporting the role of inflammatory pathways in the link between AF and cognitive impairment. This research seeks to enhance our understanding of the mechanistic relationship between AF-related inflammation and cognitive decline by synthesizing and critically appraising relevant studies.

## Method

### Protocol and registration

The systematic review protocol follows the Preferred Reporting Items for Systematic Reviews and Meta-Analysis (PRISMA) guidelines [35]. The study’s protocol was registered on Prospero under the registration number CRD4202450804. The registration link can be found in the link: https://www.crd.york.ac.uk/prospero/display_record.php?ID=CRD42024508041.

### Eligibility criteria

Eligibility criteria were determined based on the PICO guidelines. Studies meeting the following criteria were considered for inclusion: (a) adults aged 18 years and above, with a diagnosis of AF (of any classification) and cognitive decline, with or without other associated diseases; (b) studies incorporating measures of inflammation within this population; and (c) human cross-sectional and longitudinal observational studies. Exclusion criteria included: (a) Studies assessing patients with other types of arrhythmias; and (b) review articles (systematic reviews and meta-analyses), qualitative studies, case reports, book chapters, commentaries, dissertations, animal studies, and gray literature.

### Information sources and search process

A systematic search was conducted across PubMed, Web of Science, and Psych Info databases, encompassing papers published from inception to January 2024. The inclusion criteria were limited to studies available in English and Spanish. The search strategy incorporated MeSH terms related to AF, cognitive decline, and inflammation, as detailed in supplemental material 1.

### Study selection and data collection process

Studies identified through equation search were imported into Rayyan software [36]. Following the removal of duplicates, two authors independently reviewed titles and abstracts to check eligibility for inclusion. Selected papers underwent thorough text analysis by the same two authors and any discrepancies were resolved through discussion reaching an agreement. Following PRISMA guidelines, the interrater agreement was assessed, showing strong agreement as indicated by Cohen’s kappa coefficient (k = 0.89).

### Data extraction

The process of selecting studies and extracting data followed predefined inclusion and exclusion criteria. While review articles were not included, their reference lists were checked for potentially relevant sources. Data extraction involved gathering information on country of origin, year of publication, sample characteristics (i.e., number of participants, age), study design, duration of follow-up, outcome measures, assessment methods, inclusion/exclusion criteria, and reported results.

### Quality assessment

The National Heart, Lung, and Blood Institute (NHLBI) Quality Assessment Tools designed for Observational Cohort and Cross-Sectional Studies were used to assess the quality of the selected studies. This quality assessment tool comprises 14 items strategically designed to guide the author in focusing on crucial concepts to evaluate the internal validity of a study. Each item receives a rating of yes, no, not applicable, or not reported, ultimately determining the overall quality as poor, fair, or good. The evaluation process was conducted by the same independent authors, and any disparities in judgment were resolved through discussions between the two authors.

### Data analysis

Data analysis was conducted using a narrative synthesis approach, which involved presenting narrative text that summarizes the data (Table 1). This approach allows readers to evaluate outcomes while considering differences in study designs and potential sources of bias across the reviewed studies.

**Table 1:** Quality assessment scores according to the NHLBI Quality Assessment Tool for Observational Cohort and cross-sectional studies

Study	Q1	Q2	Q3	Q4	Q5	Q6	Q7	Q8	Q9	Q10	Q11	Q12	Q13	Q14	Quality Rating
Tilvis et al., [37]	Yes	Yes	Yes	Yes	No	Yes	Yes	Na	Yes	Yes	Yes	Nr	No	Yes	Good
Lappegård et al., [38]	Yes	Yes	Yes	Yes	No	Yes	Yes	Yes	Yes	Yes	Yes	Yes	No	No	Good
An and Li, [39]	Yes	Yes	NR	Yes	No	No	No	Yes	Yes	No	Yes	NR	No	No	Good
Naranjo et al., [41]	Yes	No	NR	Yes	No	No	No	Yes	Yes	No	Yes	Yes	No	Yes	Fair
Li et al., [40]	Yes	Yes	NR	Yes	No	No	No	Yes	Yes	No	Yes	NR	No	No	Good

*Note*. NA = not applicable; NR = not reported; NHLBI = National Heart, Lung, and Blood Institute

## Results

### Main findings

**Fig. 1:**
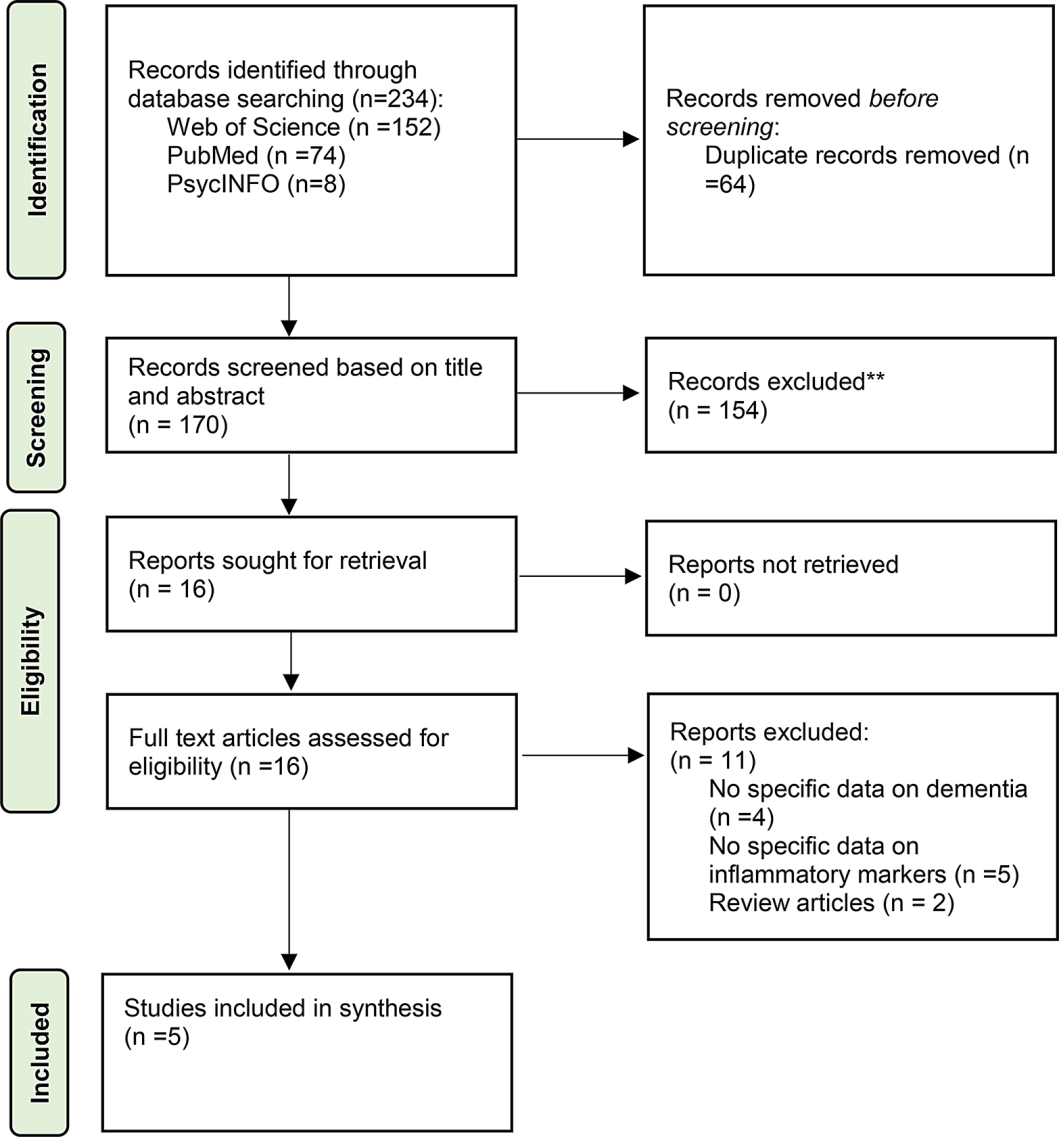
Preferred Reporting Items for Systematic Reviews and Meta-Analyses flow diagram of selected studies for inclusion

A total of 234 papers were identified from the database searches and manual reference checking. Following duplicate removal (*n* = 64), our preliminary literature search identified 170 potentially eligible studies. After screening the title and abstract, we excluded 154 studies. We then retrieved the full text of the remaining 16 studies. Of these studies, 11 were excluded, because they did not meet the inclusion criteria. Figure 1 provides a PRISMA flow diagram of the study selection process, including reasons for exclusion.

### Quality assessment

The methodological quality of all the studies was assessed using the NHLBI assessment tool (Table 2). This tool identifies flaws that could potentially impact the reported results or the study’s ability to accurately evaluate associations between exposure and outcome. Thus, of the included studies, four studies were rated as good quality [37–40], and one was considered fair quality [41].

**Table 2:** Study Design and Population characteristics

Author (year) country	Sample		Diagnosis	Inflammatory markers	Cognitive status assessment	Study design		Summary of main findings
	Sample size	Age mean (range)				Type	Time of assessment	
Tilvis et al. [37] Finland	*N* = 650	(-) (75–95)	AF	CRP	MMSE CDR	L	Baseline After 1 year After 5 years after 10 years	At baseline: Neither AF nor CRP were associated with MMSE. After 5 years: **AF and CRP predicted CDR.**
Lappegård [38] Norway	*N* = 34 treatment group = 17 placebo group = 17	Treatment group = 74.5 (-) Placebo group = 73.5 (-)	Chronic AF	MIP-1α; MIP-1β; IL-1β; IL-1RA; IL-2; IL-4; IL-5; IL-6; IL-7; IL-8; IL-9; IL-10; IL-12; IL-13; IL-15; IL-17; MCP-1; TNF-α	Multilevel Assessment Instrument MMSE Rey Auditory Verbal Learning Test Rey-Osterrieth Complex Figure Test WAIS-III: digit span WAIS-III: Vocabulary Verbal fluency Reaction time tasks (ERT, SRT, CRT) WAIS-III: digit symbol coding Stroop Color-Word Test I to III Trail-Making Test A Trail Making Test B Stroop Color-Word Test IV	L	Baseline After 1 year	For the domains speed and memory, **the correlation between the rate of decline and changes in inflammatory markers was highly significant**. There was no correlation for the domains of language, switching, and attention. **Changes in IL-1RA**,** IL-2**,** IL-9**,** IL-12**,** and MIP-1β significantly correlated with changes in cognitive functions.** For the other inflammatory markers, no statistically significant correlations were found.
An & Li [39] China	*N* = 300 Cognitive impairment = 133 No cognitive impairment = 167	Cognitive impairment = 66.14 (-) No cognitive impairment = 57.12 (-)	Combined AF	HS- CRP	MoCA	C	Hospital admission	**AF was associated with vascular cognitive impairment.** **HS- CRP was an independent risk factor of vascular cognitive impairment.**
Naranjo et al. [41] Spain	*N* = 181 MCI = 105 Controls = 76		MCI (13 with AF) Controls (1 with AF)	HS- CRP cystatin C (LpA)	MRI MMSE Free and Cued Selective Reminding Test Symbol Digit Modalities Test Letter fluency Animal fluency Rey-Osterrieth complex figure (ROCF) Recall exercises	C		**AF was associated with MCI.** **HS- CRP was not associated with MCI**.
Li et al. [40] China	*N* = 134 (NHBP = 63, AHBP = 71)		AF	HS- CRP IL-6	Tomography/MRI – Hippocampal perfusion	C		**Inflammatory biomarkers were significantly increased in patients with AF with AHBP**, in which serum **HS-CRP and IL-6 were significantly higher in Persistent AF group** than in Paroxysmal AF group. **Inflammatory markers were significantly correlated with hippocampal perfusion.** **Inflammatory markers were associated with AHBP in patients with AF.**

*Note*. AF = Atrial Fibrillation; MMSE = Mini-Mental State Examination; MoCA = Montreal Cognitive Assessment; CDR = Clinical Dementia Rating; L = longitudinal; C = cross-sectional; CRP = C-reactive protein; HS-CRP = High-sensitivity CRP; (LpA) = lipoprotein(a); CD = Cognitive Decline; MCI = Mild Cognitive impairment; NHBP = normal hippocampal blood perfusion; AHBP = abnormal hippocampal blood perfusion

### Studies characteristics

Among the five included studies, two of them were performed in the AF population [38, 40], while three studies were performed with a mixed population, i.e., cerebral infarction [39], mild cognitive impairment [41], and aged people [37]. Three of the selected studies were conducted in Europe and two in Asia. Out of all the studies analyzed, only two followed a longitudinal approach [37, 38], while the remaining three utilized a cross-sectional design.

The sample size across the studies ranged from 34 [38] to 650 [37] participants. The time of assessment ranged considerably between studies. Concerning the longitudinal studies, one study assessed outcomes one year after the initial evaluation [38], while another study conducted assessments at baseline, one year, five years, and ten years post-baseline [37]. Some studies used medical records alone for AF evaluation [37–40], while others combined medical records with electrocardiogram (ECG) diagnosis [40].

In all studies, several methods were employed to evaluate cognitive decline. For instance, Tilvis et al. [37] used the Mini-Mental State Examination (MMSE) and the Clinical Dementia Rating (CDR) to assess cognition. Cognitive decline was defined as an increase in CDR class or a decrease of ≥ 4 points in MMSE score. An and Li [39] employed the Montreal Cognitive Assessment (MoCA) and a score of ≤ 26 was classified as abnormal regarding cognition. Naranjo and colleagues [41] performed a neuropsychological and functional evaluation including the MMSE for diagnosing mild cognitive impairment (MCI). In Lappegard et al.‘s [38] study, all participants underwent a face-to-face clinical interview, followed by a comprehensive neuropsychological evaluation, that encompassed various domains (including clinical, memory, language, executive function/speed, and executive function/switching), conducted at baseline and one year after enrollment in the study. Additionally, magnetic resonance imaging (MRI) was conducted to assess changes in brain areas. At last, Li et al., [40] used computed tomography to measure hippocampal perfusion to assess cognitive impairment.

In all studies, participants went through clinical evaluations, and blood samples were collected to check inflammatory markers. C-reactive protein (CRP) was measured in all studies. High-sensitivity C-reactive protein (hs-CRP), fibroblast growth factor (FGF), granulocyte colony-stimulating factor (G-CSF), granulocyte-macrophage colony-stimulating factor (GM-CSF), interleukin-1 receptor antagonist (IL-1RA), interleukin (IL)-2, IL-9, IL-12, IL-13, IL-17, interferon-γ (IFNγ) and macrophage inflammatory protein-1β (MIP-1β) were variables included in the study of Lappegard et al. [38].

### Study findings

Regarding study results, the association between AF and cognitive decline, and between inflammatory processes and cognitive decline were found in two studies with the AF population [38, 40], and in three studies with mixed populations with AF (aged people, cerebral infarction, and mild cognitive impairment) [37, 39, 41].

#### AF population studies

In a clinical trial that examines the relationship between neurocognitive functions and inflammatory burden in older patients with AF, the results showed a statistically significant correlation between reduction in several inflammatory markers and delayed neurocognitive decline (especially memory and speed of information processing) as well as a reduced volume loss in certain cerebral areas (i.e., hippocampus and left amygdala) [38]. Additionally, another study revealed a significant correlation between inflammation and hippocampal perfusion in patients with AF, indicating that elevated levels of inflammatory markers were associated with alterations in hippocampal perfusion [40].

#### Mix population studies

Concerning the mixed population studies, the current systematic review focused exclusively on the results from individuals diagnosed with AF. The coexistence of AF diagnosis and elevated hs-CRP concentrations were significantly associated with cognitive impairment in individuals suffering from cerebral infarction with a multivariate model approach [39]. In this study, hs-CRP emerged as a predictive risk factor for cognitive impairment although this association needs further confirmation in cerebral infarction patients.

In a longitudinal study with aged individuals, results showed that cognitive decline (measured by MMSE and CDR) was predicted by the presence of AF and elevated CRP, only in the 5-year follow-up [37]. At baseline and 10 years, neither the presence of AF nor the elevated CRP were associated with cognitive decline. Furthermore, the presence of AF was significantly associated with an increased risk of MCI but no relationship was found between hs-CPR levels and cognitive impairment [41].

## Discussion

This systematic review is the first to focus exclusively on the inflammatory mechanisms underlying the association between AF and cognitive decline. Despite the limited body of research in this area, the findings highlight inflammation as a potential mediator, underscoring the need for randomized controlled trials to address the gaps in observational evidence. Additionally, variability in cognitive assessment methodologies likely contributes to inconsistencies in the reported associations between AF and cognitive decline. Nevertheless, the five included studies provide valuable insights into the complex association between inflammation, AF, and cognitive decline.

The pathophysiology underlying the link between AF and cognitive decline remains a critical knowledge gap. Addressing this gap is essential to mitigate cognitive decline and develop targeted interventions for the AF population. All studies included in this review, report an association between AF and cognitive decline [37–42], observed not only in older adults [37] but also in individuals with comorbidities such as cerebral infarction [39] and mild cognitive impairment (MCI) [41]. Longitudinal evidence underscores the role of early-onset AF and prolonged AF duration in accelerating cognitive decline and dementia [43], potentially due to neuronal damage and blood-brain barrier disruption [44]. Both conditions are linked to a pro-inflammatory state [4, 29], with systemic inflammation markers inversely associated with cognitive performance in a large population-based study [29]. Inflammation also plays a critical role in AF-related atrial remodeling [45–47], further implicating its potential mediator in this relationship.

In AF, inflammation plays a dual role: it can be triggered by arrhythmogenic risk factors leading to cardiac remodeling [27, 47], and AF itself can induce inflammation through thromboembolic events and endothelial damage, resulting in immune cell infiltration [27, 47]. Chronic low-grade inflammation contributes to atrial remodeling, collagen deposition, and fatty tissue accumulation [48–53]. Additionally, the NOD-like receptor protein 3 (NLRP3) inflammasome activation has been implicated in both AF and its associated cardiovascular diseases [54–61] resulting in expression, maturation, and release of potent inflammatory mediators. Elevated levels of C-reactive protein (CRP), a widely recognized inflammatory marker, are associated with AF onset, progression, and adverse outcomes, particularly when combined with interleukin-6 (IL-6) [62–67]. In the context of cognitive decline, CRP and other inflammatory markers are linked to an increased risk of dementia [68–74], with amyloid-beta plaques contributing to neuroinflammation and neurodegeneration in Alzheimer’s disease [69, 71]. These findings suggest that inflammation mediates the connection between AF and cognitive decline [4, 29, 74].

Evidence from neuroimaging and intervention studies further supports the role of inflammation in AF-related cognitive decline [25, 75, 76]. Lipid-lowering therapies that reduce inflammatory markers have shown promise in improving cognitive outcomes, suggesting a therapeutic link between inflammation control and neuroprotection [38, 77, 78]. However, contradictory findings, such as the absence of a correlation between hs-CRP and cognitive impairment in one study [41], highlight the multifactorial nature of this relationship and the influence of confounding factors, such as obesity [41, 79–82], also an inflammatory condition [82–84].

Emerging research points to novel inflammatory pathways, including the NLRP3 inflammasome, as potential therapeutic targets [85–89]. Advances in single-cell RNA sequencing have identified specific macrophage subsets involved in AF-associated inflammation, offering promising avenues for immunotherapy [90]. Anti-inflammatory interventions may complement traditional AF management by mitigating both arrhythmia and its cognitive consequences [85–89]. However, translating these findings into clinical practice requires identifying reliable biomarkers, optimizing intervention timing, and addressing the bidirectional relationship between systemic inflammation and AF [78, 91].

Thus, cognitive decline should be considered a key outcome variable in longitudinal, well-designed prospective studies to explore the complex pathways by which AF impacts cognitive function. Such studies will not only contribute to a deeper understanding of the underlying pathophysiological mechanisms but also enable the development of targeted interventions [92]. Given the systemic nature of AF, determining the causal relationship between systemic immune responses and AF is challenging, as these processes may exacerbate each other [93, 94].

Additionally, it is important to emphasize that cognitive assessment in patients with AF presents unique challenges, such as the precision of cognitive assessments being inversely correlated with the number of patients included in studies [37–41]. This pattern reflects a limitation in study designs, where smaller cohorts may allow for more comprehensive cognitive evaluations [e.g. 38], thereby uncovering early-stage impairments. Contrarily, larger-scale studies may prioritize broader population analyses [e.g. 37], potentially underestimating cognitive deficits. From a clinical perspective, this underscores the importance of systematically assessing cognitive function in AF patients [4]. Early detection through standardized protocols would not only allow for timely interventions but would also contribute to a deeper understanding of the relationship between AF and cognitive decline. Therefore, incorporating cognitive assessments into clinical practice for AF patients is crucial to addressing this knowledge gap and improving patient outcomes [4].

Understanding the association between AF and cognitive decline, and the underlying inflammatory mechanisms, has important implications for clinical practice, research priorities, and public health strategies. Insights from this systematic review may inform risk stratification, preventive interventions, and personalized management approaches for individuals with AF at risk of cognitive decline. Furthermore, identifying inflammatory pathways implicated in AF-related cognitive decline could offer new therapeutic paths, potentially improving clinical outcomes and QoL for AF patients.

### Limitations and future directions

To effectively address the cognitive decline in patients with AF, it is crucial to understand the factors that contribute to these conditions [95]. This knowledge can significantly reduce the overall burden of associated health issues. While this systematic review provides up-to-date findings from current literature on the association between AF, cognitive decline, and inflammatory processes, several limitations should be acknowledged. In fact, the few studies included in the review, particularly in the context of the AF population, may limit the generalization of the findings. Moreover, the heterogeneity among the included studies, such as differences in participant characteristics, and study designs, introduces potential sources of bias and variability. For instance, the mixed population studies encompass diverse groups, including aged individuals, those with cerebral infarction, and individuals with mild cognitive impairment, which may confound the observed associations. Additionally, the reliance on cross-sectional designs limits the ability to establish causal relationships between AF, inflammatory markers, and cognitive decline. Furthermore, some studies only measured cognitive decline using instruments such as MMSE and CDR, which may not capture the full spectrum of cognitive impairment. CRP emerges as the inflammatory marker with the most substantial evidence in the context of AF and cognitive decline and is often the only marker assessed in many studies. As a well-established marker of systemic inflammation, CRP is a surrogate for the broader inflammatory processes, offering a practical tool for exploring these intricate relationships [4]. CRP has been extensively studied in cardiovascular and neurological research, providing a robust foundation for investigating its role in AF and cognitive decline. Previous studies have revealed associations between elevated CRP levels and increased risk of both AF and cognitive impairment, reinforcing its utility as a primary marker. So, it is a widely recognized and well-established biomarker used to assess systemic inflammation. Its utility is underscored by its ease of measurement, cost-effectiveness, and standardized application across clinical settings, making it a practical choice for research and routine healthcare [98]. The easy availability of data facilitates the use of this biomarker: many existing large cohort studies or clinical trials include CRP measurements as part of routine biomarker panels, making it readily available for retrospective analysis. Other inflammatory markers, such as interleukins (e.g., IL-6) or tumor necrosis factor-alpha (TNF-α), are less commonly measured in large-scale studies due to higher costs or logistical challenges. In addition, studying CRP alone is simpler since studying additional inflammatory markers may require more invasive procedures or higher costs, which can limit feasibility, especially in large populations or in elderly patients with a tendency towards cognitive decline. Thus, using CRP as a single marker reduces complexity and, at the same time, provides significant information about the inflammation hypothesis. However, despite its utility, CRP alone may not fully capture the complexity of the inflammatory processes linking AF and cognitive decline. Other markers, such as IL-6, TNF-α, or fibrinogen, could provide complementary insights into specific inflammatory pathways. Future studies might benefit from a multi-marker approach to enhance understanding of the underlying mechanisms. Also, while the systematic review highlights promising associations between AF, inflammation, and cognitive decline, there is a need for larger, well-designed prospective studies to clarify the underlying inflammatory mechanisms and clinical implications of these relationships. Furthermore, the findings from this study are expected to inform cognitive decline risk stratification, and future research directions, guiding the development of targeted interventions aimed at preserving cognitive function and improving outcomes in patients with AF.

## Conclusion

We hypothesized an inflammatory bridge between AF and cognitive decline. Although few studies were included, the findings presented in the systematic review have significant implications for both clinical practice and future research efforts. Firstly, the observed associations between AF, inflammatory markers, and cognitive decline underscore the potential importance of considering inflammatory pathways in the management of cognitive decline, in this population. The significant correlation between reduction in inflammatory markers and delayed neurocognitive decline in older patients with AF suggests that targeting inflammation may represent a promising therapeutic approach for mitigating cognitive decline in AF population. Furthermore, the association between AF and cognitive decline, particularly in aged individuals and those with cerebral infarction, highlights the need for increased awareness and screening for cognitive impairment in patients with AF, as well as closer monitoring of inflammatory markers to identify individuals at higher risk [92, 96, 97]. These findings suggest that addressing both AF and inflammation may have synergistic effects in preserving cognitive function and reducing the burden of cognitive decline in vulnerable populations. In addition, recognizing inflammation’s role may refine risk stratification strategies and inform personalized interventions to preserve cognitive function in individuals with AF. Thus, regular monitoring of biomarkers is an important tool in assessing intervention effectiveness, allowing treatment to be adjusted as necessary, offering a personalized and proactive approach to managing AF and preventing cognitive decline [91, 92]. As a result, inflammatory markers in early diagnosis may improve patient’s QoL and significantly reduce the cognitive impact associated with AF [92].

This systematic review highlights the need for further research to elucidate the relationship between inflammation, atrial fibrillation (AF), and cognitive decline. Advancing understanding in this area is essential for developing strategies to delay the onset of cognitive impairment, reduce the risk of dementia, and ultimately improve clinical management and outcomes for individuals with AF.

## Electronic Supplementary Material

Below is the link to the electronic supplementary material.

Supplementary Material 1

## References

[1] Kornej J, Börschel CS, Benjamin EJ, Schnabel RB (2020) Epidemiology of Atrial Fibrillation in the 21st Century: Novel methods and New insights. Circul Res 127(1):4–20. 10.1161/CIRCRESAHA.120.316340 10.1161/CIRCRESAHA.120.316340 PMC7577553 32716709

[2] Benjamin EJ, Muntner P, Alonso A et al (2020) Heart disease and stroke statistics-2019 update: A report from the american heart association [published correction appears in circulation.;141(2):e33. 10.1161/CIR.0000000000000659 10.1161/CIR.0000000000000746 31928433

[3] Dilaveris PE, Kennedy HL (2017) Silent atrial fibrillation: epidemiology, diagnosis, and clinical impact. Clin Cardiol 40(6):413–418. 10.1002/clc.22667 28273368 10.1002/clc.22667 PMC6490532

[4] Rivard L, Friberg L, Conen D et al (2022) Atrial fibrillation and dementia: a Report from the AF-SCREEN International collaboration. Circulation 145(5):392–409. 10.1161/CIRCULATIONAHA.121.055018 35100023 10.1161/CIRCULATIONAHA.121.055018

[5] Bunch TJ, Weiss JP, Crandall BG et al (2010) Atrial fibrillation is independently associated with senile, vascular, and Alzheimer’s dementia. Heart Rhythm 7(4):433–437. 10.1016/j.hrthm.2009.12.004 20122875 10.1016/j.hrthm.2009.12.004

[6] Chen LY, Norby FL, Gottesman RF et al (2018) Association of Atrial Fibrillation with Cognitive Decline and Dementia over 20 years: the ARIC-NCS (atherosclerosis risk in communities Neurocognitive Study). J Am Heart Assoc 7(6):e007301. 10.1161/JAHA.117.007301 29514809 10.1161/JAHA.117.007301 PMC5907543

[7] de Bruijn RF, Heeringa J, Wolters FJ et al (2015) Association between Atrial Fibrillation and Dementia in the General Population. JAMA Neurol 72(11):1288–1294. 10.1001/jamaneurol.2015.2161 26389654 10.1001/jamaneurol.2015.2161

[8] Islam MM, Poly TN, Walther BA et al (2019) Association between Atrial Fibrillation and Dementia: a Meta-analysis. Front Aging Neurosci 11:305. 10.3389/fnagi.2019.00305 31780919 10.3389/fnagi.2019.00305 PMC6857071

[9] Kalantarian S, Stern TA, Mansour M, Ruskin JN (2013) Cognitive impairment associated with atrial fibrillation: a meta-analysis. Ann Intern Med 158(5 Pt 1):338–346. 10.7326/0003-4819-158-5-201303050-00007 23460057 10.7326/0003-4819-158-5-201303050-00007 PMC4465526

[10] Kwok CS, Loke YK, Hale R, Potter JF, Myint PK (2011) Atrial fibrillation and incidence of dementia: a systematic review and meta-analysis. Neurology 76(10):914–922. 10.1212/WNL.0b013e31820f2e3811 21383328 10.1212/WNL.0b013e31820f2e38

[11] Leszek J, Mikhaylenko EV, Belousov DM et al (2021) The links between Cardiovascular diseases and Alzheimer’s Disease. Curr Neuropharmacol 19(2):152–169. 10.2174/1570159X18666200729093724 32727331 10.2174/1570159X18666200729093724 PMC8033981

[12] Liu DS, Chen J, Jian WM, Zhang GR, Liu ZR (2019) The association of atrial fibrillation and dementia incidence: a meta-analysis of prospective cohort studies. J Geriatr Cardiol 16(3):298–306. 10.11909/j.issn.1671-5411.2019.03.006 31080473 10.11909/j.issn.1671-5411.2019.03.006 PMC6500564

[13] Marzona I, O’Donnell M, Teo K et al (2012) Increased risk of cognitive and functional decline in patients with atrial fibrillation: results of the ONTARGET and TRANSCEND studies. CMAJ 184(6):E329–E336. 10.1503/cmaj.111173 22371515 10.1503/cmaj.111173 PMC3314061

[14] Papanastasiou CA, Theochari CA, Zareifopoulos N et al (2021) Atrial fibrillation is Associated with cognitive impairment, all-cause dementia, vascular dementia, and Alzheimer’s Disease: a systematic review and Meta-analysis. J Gen Intern Med 36(10):3122–3135. 10.1007/s11606-021-06954-815 34244959 10.1007/s11606-021-06954-8 PMC8481403

[15] Saglietto A, Matta M, Gaita F, Jacobs V, Bunch TJ, Anselmino M (2019) Stroke-independent contribution of atrial fibrillation to dementia: a meta-analysis. Open Heart 6(1):e000984 Published 2019 May 2. 10.1136/openhrt-2018-000984 31217998 10.1136/openhrt-2018-000984 PMC6546265

[16] Santangeli P, Di Biase L, Bai R et al (2012) Atrial fibrillation and the risk of incident dementia: a meta-analysis. Heart Rhythm 9(11):1761–1768. 10.1161/JAHA.122.025653 22863685 10.1016/j.hrthm.2012.07.026

[17] Magnani JW, Rienstra M, Lin H et al (2011) Atrial fibrillation: current knowledge and future directions in epidemiology and genomics. Circulation 124(18):1982–1993. 10.1161/CIRCULATIONAHA.111.039677 22042927 10.1161/CIRCULATIONAHA.111.039677 PMC3208425

[18] Horstmann S, Rizos T, Rauch G, Fuchs M, Arden C, Veltkamp R (2014) Atrial fibrillation and prestroke cognitive impairment in stroke. J Neurol 261(3):546–553. 10.1007/s00415-013-7233-3 24413641 10.1007/s00415-013-7233-3

[19] Blum S, Conen D (2023) Mechanisms and clinical manifestations of Cognitive decline in Atrial Fibrillation patients: potential implications for preventing dementia. Can J Cardiol 39(2):159–171. 10.1016/j.cjca.2022.10.013 36252904 10.1016/j.cjca.2022.10.013

[20] Ding M, Qiu C, Atrial, Fibrillation (2018) Cognitive decline, and dementia: an epidemiologic review. Curr Epidemiol Rep 5(3):252–261. 10.1007/s40471-018-0159-7 30148041 10.1007/s40471-018-0159-7 PMC6096854

[21] Manolis TA, Manolis AA, Apostolopoulos EJ, Melita H, Manolis AS (2020) Atrial fibrillation and cognitive impairment: an Associated Burden or Burden by Association? Angiology 71(6):498–519. 10.1177/0003319720910669 32233780 10.1177/0003319720910669

[22] Dagres N, Chao TF, Fenelon G et al (2018) European Heart Rhythm Association (EHRA)/Heart Rhythm Society (HRS)/Asia Pacific Heart Rhythm Society (APHRS)/Latin American Heart Rhythm Society (LAHRS) expert consensus on arrhythmias and cognitive function: what is the best practice? Europace 20(9):1399–1421. 10.1093/europace/euy046 29562326 10.1093/europace/euy046 PMC6658813

[23] Sepehri Shamloo A, Dagres N, Müssigbrodt A et al (2020) Atrial fibrillation and cognitive impairment: New insights and future directions. Heart Lung Circ 29(1):69–85. 10.1016/j.hlc.2019.05.185 31262618 10.1016/j.hlc.2019.05.185

[24] Carbone G, Ercolano E, Bencivenga L et al (2024) Atrial fibrillation and dementia: Focus on Shared Pathophysiological mechanisms and therapeutic implications. J Am Med Dir Assoc 25(3):465–469. 10.1016/j.jamda.2024.01.010 38359898 10.1016/j.jamda.2024.01.010

[25] Madhavan M, Graff-Radford J, Piccini JP, Gersh BJ (2018) Cognitive dysfunction in atrial fibrillation. Nat Rev Cardiol 15(12):744–756. 10.1038/s41569-018-0075-z 30275499 10.1038/s41569-018-0075-z

[26] Dietzel J, Haeusler KG, Endres M (2018) Does atrial fibrillation cause cognitive decline and dementia? Europace 20(3):408–419. 10.1093/europace/eux031 28387847 10.1093/europace/eux031

[27] Ihara K, Sasano T (2022) Role of inflammation in the pathogenesis of Atrial Fibrillation. Front Physiol 13:862164. 10.3389/fphys.2022.862164 35492601 10.3389/fphys.2022.862164 PMC9047861

[28] Korantzopoulos P, Letsas KP, Tse G, Fragakis N, Goudis CA, Liu T (2018) Inflammation and atrial fibrillation: a comprehensive review. J Arrhythm 34(4):394–401. 10.1002/joa3.12077 30167010 10.1002/joa3.12077 PMC6111477

[29] Guo Z, Zheng Y, Geng J et al (2024) Unveiling the link between systemic inflammation markers and cognitive performance among older adults in the US: a population-based study using NHANES 2011–2014 data. J Clin Neurosci 119:45–51. 10.1016/j.jocn.2023.11.004 37979310 10.1016/j.jocn.2023.11.004

[30] Sartori AC, Vance DE, Slater LZ, Crowe M (2012) The impact of inflammation on cognitive function in older adults: implications for healthcare practice and research. J Neurosci Nurs 44(4):206–217. 10.1097/JNN.0b013e3182527690 22743812 10.1097/JNN.0b013e3182527690 PMC3390758

[31] Alonso A, Arenas de Larriva AP (2016) Atrial fibrillation, cognitive decline and Dementia. Eur Cardiol 11(1):49–53. 10.15420/ecr.2016:13:2 27547248 10.15420/ecr.2016:13:2 PMC4988519

[32] Alfaddagh A, Martin SS, Leucker TM et al (2020) Inflammation and cardiovascular disease: from mechanisms to therapeutics. Am J Prev Cardiol 4:100130 Published 2020 Nov 21. 10.1016/j.ajpc.2020.100130 34327481 10.1016/j.ajpc.2020.100130 PMC8315628

[33] Gilhus NE, Deuschl G (2019) Neuroinflammation - a common thread in neurological disorders. Nat Rev Neurol 15(8):429–430. 10.1038/s41582-019-0227-8 31263256 10.1038/s41582-019-0227-8

[34] Iannucci J, Renehan W, Grammas P (2020) Thrombin, a Mediator of Coagulation, Inflammation, and Neurotoxicity at the Neurovascular Interface: Implications for Alzheimer’s Disease. *Front Neurosci*.;14:762. Published 2020 Jul 24. 10.3389/fnins.2020.00762 10.3389/fnins.2020.00762 PMC7393221 32792902

[35] Moher D, Liberati A, Tetzlaff J, Altman DG, PRISMA Group (2009) Preferred reporting items for systematic reviews and meta-analyses: the PRISMA statement. PLoS Med 6(7):e1000097. 10.1371/journal.pmed.1000097 19621072 10.1371/journal.pmed.1000097 PMC2707599

[36] Ouzzani M, Hammady H, Fedorowicz Z, Elmagarmid A (2016) Rayyan-a web and mobile app for systematic reviews. Syst Rev 5(1):210 Published 2016 Dec 5. 10.1186/s13643-016-0384-4 27919275 10.1186/s13643-016-0384-4 PMC5139140

[37] Tilvis RS, Kähönen-Väre MH, Jolkkonen J, Valvanne J, Pitkala KH, Strandberg TE (2004) Predictors of cognitive decline and mortality of aged people over a 10-year period. J Gerontol Biol Sci Med Sci 59(3):268–274. 10.1093/gerona/59.3.m268 10.1093/gerona/59.3.m268 15031312

[38] Lappegård KT, Pop-Purceleanu M, van Heerde W, Sexton J, Tendolkar I, Pop G (2013) Improved neurocognitive functions correlate with reduced inflammatory burden in atrial fibrillation patients treated with intensive cholesterol lowering therapy. J Neuroinflammation 10:78. 10.1186/1742-2094-10-78 23809138 10.1186/1742-2094-10-78 PMC3699385

[39] An XL, Li CL (2015) Analysis of risk factors for vascular cognitive impairment in patients with cerebral infarction. Cell Biochem Biophys 71(2):673–677. 10.1007/s12013-014-0246-4 25227943 10.1007/s12013-014-0246-4

[40] Li J, Liang G, Huang F et al (2023) The correlation of inflammation, oxidative stress, and hippocampal perfusion in patients with Atrial Fibrillation. Int Heart J 64(6):1018–1024. 10.1536/ihj.23-051 38030288 10.1536/ihj.23-051

[41] Casado Naranjo I, Portilla Cuenca JC, Duque de San Juan B et al (2015) Association of vascular factors and amnestic mild cognitive impairment: a comprehensive approach. J Alzheimers Dis 44(2):695–704. 10.3233/JAD-141770 25362037 10.3233/JAD-141770

[42] Zhang MJ, Norby FL, Lutsey PL et al (2019) Association of Left Atrial Enlargement and Atrial Fibrillation with cognitive function and decline: the ARIC-NCS. J Am Heart Assoc 8(23):e013197. 10.1161/JAHA.119.013197 31766970 10.1161/JAHA.119.013197 PMC6912953

[43] Singh-Manoux A, Fayosse A, Sabia S et al (2017) Atrial fibrillation as a risk factor for cognitive decline and dementia. Eur Heart J 38(34):2612–2618. 10.1093/eurheartj/ehx208 28460139 10.1093/eurheartj/ehx208 PMC5837240

[44] Galenko O, Jacobs V, Knight S et al (2019) Circulating levels of biomarkers of Cerebral Injury in patients with Atrial Fibrillation. Am J Cardiol 124(11):1697–1700. 10.1016/j.amjcard.2019.08.027 31575426 10.1016/j.amjcard.2019.08.027

[45] Quigley A, MacKay-Lyons M, Eskes G (2020) Effects of Exercise on Cognitive performance in older adults: a narrative review of the evidence, possible Biological mechanisms, and recommendations for Exercise prescription. J Aging Res 2020:1407896 Published 2020 May 14. 10.1155/2020/1407896 32509348 10.1155/2020/1407896 PMC7244966

[46] Mezzaroma E, Abbate A, Toldo S (2021) The inflammasome in heart failure. Curr Opin Physiol 19:105–112. 10.1016/j.cophys.2020.09.013 34917871 10.1016/j.cophys.2020.09.013 PMC8670733

[47] Roka A, Burright I (2023) Remodeling in Persistent Atrial Fibrillation: pathophysiology and therapeutic Targets—A systematic review. Physiologia 3(1):43–72. 10.3390/physiologia3010004

[48] Frustaci A, Chimenti C, Bellocci F, Morgante E, Russo MA, Maseri A (1997) Histological substrate of atrial biopsies in patients with lone atrial fibrillation. Circulation 96(4):1180–1184. 10.1161/01.cir.96.4.1180 9286947 10.1161/01.cir.96.4.1180

[49] Barcena ML, Aslam M, Pozdniakova S, Norman K, Ladilov Y (2022) Cardiovascular Inflammaging: mechanisms and translational aspects. Cells 11(6):1010. 10.3390/cells11061010 35326461 10.3390/cells11061010 PMC8946971

[50] Batal O, Schoenhagen P, Shao M et al (2010) Left atrial epicardial adiposity and atrial fibrillation. Circ Arrhythm Electrophysiol 3(3):230–236. 10.1161/CIRCEP.110.957241 20504944 10.1161/CIRCEP.110.957241 PMC2974566

[51] Takahashi Y, Yamaguchi T, Otsubo T et al (2023) Histological validation of atrial structural remodelling in patients with atrial fibrillation. Eur Heart J 44(35):3339–3353. 10.1093/eurheartj/ehad396 37350738 10.1093/eurheartj/ehad396 PMC10499545

[52] Costantini O (2019) Basic principles of Cardiac Electrophysiology. Med Clin North Am 103(5):767–774. 10.1016/j.mcna.2019.04.002 31378323 10.1016/j.mcna.2019.04.002

[53] Tellez JO, Mczewski M, Yanni J et al (2011) Ageing-dependent remodelling of ion channel and Ca2 + clock genes underlying sino-atrial node pacemaking. Exp Physiol 96(11):1163–1178. 10.1113/expphysiol.2011.057752 21724736 10.1113/expphysiol.2011.057752

[54] Dobrev D, Heijman J, Hiram R, Li N, Nattel S (2023) Inflammatory signalling in atrial cardiomyocytes: a novel unifying principle in atrial fibrillation pathophysiology. Nat Rev Cardiol 20(3):145–167. 10.1038/s41569-022-00759-w 36109633 10.1038/s41569-022-00759-w PMC9477170

[55] Toldo S, Abbate A (2018) The NLRP3 inflammasome in acute myocardial infarction. Nat Rev Cardiol 15(4):203–214. 10.1038/nrcardio.2017.161 29143812 10.1038/nrcardio.2017.161

[56] Abbate A et al (2020) Interleukin-1 and the inflammasome as therapeutic targets in cardiovascular disease. Circul Res 126(9):1260–1280 10.1161/CIRCRESAHA.120.315937 PMC8760628 32324502

[57] Bauernfeind FG, Horvath G, Stutz A et al (2009) Cutting edge: NF-kappaB activating pattern recognition and cytokine receptors license NLRP3 inflammasome activation by regulating NLRP3 expression. J Immunol 183(2):787–791. 10.4049/jimmunol.0901363 19570822 10.4049/jimmunol.0901363 PMC2824855

[58] Fender AC, Kleeschulte S, Stolte S et al (2020) Thrombin receptor PAR4 drives canonical NLRP3 inflammasome signaling in the heart. Basic Res Cardiol 115(2):10. 10.1007/s00395-019-0771-9 31912235 10.1007/s00395-019-0771-9 PMC7384378

[59] Suetomi T, Willeford A, Brand CS et al (2018) Inflammation and NLRP3 inflammasome activation initiated in response to pressure overload by Ca^2+^/Calmodulin-Dependent protein kinase II δ signaling in Cardiomyocytes are essential for adverse Cardiac Remodeling. Circulation 138(22):2530–2544. 10.1161/CIRCULATIONAHA.118.034621 30571348 10.1161/CIRCULATIONAHA.118.034621 PMC6309790

[60] Gong T, Yang Y, Jin T, Jiang W, Zhou R (2018) Orchestration of NLRP3 inflammasome activation by Ion Fluxes. Trends Immunol 39(5):393–406. 10.1016/j.it.2018.01.009 29452983 10.1016/j.it.2018.01.009

[61] Tschopp J, Schroder K (2010) NLRP3 inflammasome activation: the convergence of multiple signalling pathways on ROS production? Nat Rev Immunol 10(3):210–215. 10.1038/nri2725 20168318 10.1038/nri2725

[62] Chung MK, Martin DO, Sprecher D et al (2001) C-reactive protein elevation in patients with atrial arrhythmias: inflammatory mechanisms and persistence of atrial fibrillation. Circulation 104(24):2886–2891. 10.1161/hc4901.101760 11739301 10.1161/hc4901.101760

[63] Galea R, Cardillo MT, Caroli A et al (2014) Inflammation and C-reactive protein in atrial fibrillation: cause or effect? *Tex Heart Inst J*.;41(5):461–468. Published 2014 Oct 1. 10.14503/THIJ-13-3466 10.14503/THIJ-13-3466 PMC4189345 25425976

[64] Ozkan E, Elcik D, Barutcu S et al (2023) Inflammatory markers as predictors of Atrial Fibrillation recurrence: exploring the C-Reactive protein to albumin ratio in Cryoablation patients. J Clin Med 12(19):6313. 10.3390/jcm12196313 37834958 10.3390/jcm12196313 PMC10573371

[65] Conway DS, Buggins P, Hughes E, Lip GY (2004) Relationship of interleukin-6 and C-reactive protein to the prothrombotic state in chronic atrial fibrillation. J Am Coll Cardiol 43(11):2075–2082. 10.1016/j.jacc.2003.11.062 15172416 10.1016/j.jacc.2003.11.062

[66] Hadi HA, Alsheikh-Ali AA, Mahmeed WA, Suwaidi JM (2010) Inflammatory cytokines and atrial fibrillation: current and prospective views. J Inflamm Res 3:75–97. 10.2147/JIR.S10095 22096359 10.2147/JIR.S10095 PMC3218735

[67] Held C, White HD, Stewart RAH et al (2017) Inflammatory biomarkers Interleukin-6 and C-Reactive protein and outcomes in stable Coronary Heart Disease: experiences from the STABILITY (stabilization of atherosclerotic plaque by Initiation of Darapladib Therapy) Trial. J Am Heart Assoc 6(10):e005077. 10.1161/JAHA.116.005077 29066452 10.1161/JAHA.116.005077 PMC5721818

[68] Merighi S, Nigro M, Travagli A, Gessi S (2022) Microglia and Alzheimer’s Disease. Int J Mol Sci 23(21):12990 Published 2022 Oct 27. 10.3390/ijms232112990 36361780 10.3390/ijms232112990 PMC9657945

[69] Hampel H, Hardy J, Blennow K et al (2021) The Amyloid-β pathway in Alzheimer’s Disease. Mol Psychiatry 26(10):5481–5503. 10.1038/s41380-021-01249-0 34456336 10.1038/s41380-021-01249-0 PMC8758495

[70] Wang WY, Tan MS, Yu JT, Tan L (2015) Role of pro-inflammatory cytokines released from microglia in Alzheimer’s disease. Ann Transl Med 3(10):136. 10.3978/j.issn.2305-5839.2015.03.49 26207229 10.3978/j.issn.2305-5839.2015.03.49 PMC4486922

[71] Sehar U, Rawat P, Reddy AP, Kopel J, Reddy PH (2022) Amyloid Beta in aging and Alzheimer’s Disease. Int J Mol Sci 23(21):12924. 10.3390/ijms232112924 36361714 10.3390/ijms232112924 PMC9655207

[72] Xie J, Van Hoecke L, Vandenbroucke RE (2022) The impact of systemic inflammation on Alzheimer’s Disease Pathology. Front Immunol 12:796867. 10.3389/fimmu.2021.796867 35069578 10.3389/fimmu.2021.796867 PMC8770958

[73] Domingues C, da Cruz E, Silva OAB, Henriques AG (2017) Impact of cytokines and chemokines on Alzheimer’s Disease Neuropathological Hallmarks. Curr Alzheimer Res 14(8):870–882. 10.2174/1567205014666170317113606 28317487 10.2174/1567205014666170317113606 PMC5543563

[74] Choubey U, Bansal V, Shah P et al (2023) Atrial fibrillation and dementia: not just a coincidence. J Geriatr Cardiol 20(9):697–701. 10.26599/1671-5411.2023.09.003 37840632 10.26599/1671-5411.2023.09.003 PMC10568548

[75] Silva DS, Coan AC, Avelar WM (2019) Neuropsychological and neuroimaging evidences of cerebral dysfunction in stroke-free patients with atrial fibrillation: a review. J Neurol Sci 399:172–181. 10.1016/j.jns.2019.02.027 30825695 10.1016/j.jns.2019.02.027

[76] Yu AYX, Coutts SB (2018) Role of Brain and Vessel Imaging for the evaluation of transient ischemic attack and minor stroke. Stroke 49(7):1791–1795. 10.1161/STROKEAHA.118.016618 29880552 10.1161/STROKEAHA.118.016618

[77] van Kuilenburg J, Lappegård K, Sexton J et al (2011) Persisting thrombin activity in elderly patients with atrial fibrillation on oral anticoagulation is decreased by anti-inflammatory therapy with intensive cholesterol-lowering treatment. J Clin Lipidol 5(4):273–280. 10.1016/j.jacl.2011.05.003 21784372 10.1016/j.jacl.2011.05.003

[78] Bodagh N, Kotadia I, Gharaviri A et al (2023) The impact of Atrial Fibrillation treatment strategies on cognitive function. J Clin Med 12(9):3050. 10.3390/jcm12093050 37176490 10.3390/jcm12093050 PMC10179566

[79] Miralbell J, López-Cancio E, López-Oloriz J et al (2013) Cognitive patterns in relation to biomarkers of cerebrovascular disease and vascular risk factors. Cerebrovasc Dis 36(2):98–105. 10.1159/000352059 24029412 10.1159/000352059

[80] Zeng Q et al (2019) Correlations of serum cystatin C level and gene polymorphism with vascular cognitive impairment after acute cerebral infarction. Neurol Sci 40:1049–1054. 10.1007/s10072-019-03777-8 30805744 10.1007/s10072-019-03777-8

[81] Jenny NS, French B, Arnold AM et al (2012) Long-term assessment of inflammation and healthy aging in late life: the Cardiovascular Health Study all stars. J Gerontol Biol Sci Med Sci 67(9):970–976. 10.1093/gerona/glr261 10.1093/gerona/glr261 PMC3436091 22367431

[82] Leite F, Lima M, Marino F, Cosentino M, Ribeiro L (2016) Dopaminergic receptors and tyrosine hydroxylase expression in Peripheral Blood mononuclear cells: a distinct pattern in central obesity. PLoS ONE 11(1):e0147483. 10.1371/journal.pone.0147483 26808524 10.1371/journal.pone.0147483 PMC4726756

[83] Koh YH, Lew LZW, Franke KB et al (2022) Predictive role of atrial fibrillation in cognitive decline: a systematic review and meta-analysis of 2.8 million individuals. Europace 24(8):1229–1239. 10.1093/europace/euac003 35061884 10.1093/europace/euac003 PMC9435641

[84] Ang YS, Rajamani S, Haldar SM, Hüser J (2020) A New Therapeutic Framework for Atrial Fibrillation Drug Development. Circ Res 127(1):184–201. 10.1161/CIRCRESAHA.120.316576 32717173 10.1161/CIRCRESAHA.120.316576

[85] Bretto E, Ribaldone DG, Caviglia GP, Saracco GM, Bugianesi E, Frara S (2023) Inflammatory bowel disease: emerging therapies and future treatment strategies. Biomedicines 11(8):2249. 10.3390/biomedicines11082249 37626745 10.3390/biomedicines11082249 PMC10452708

[86] Bouras G, Giannopoulos G, Hatzis G, Alexopoulos D, Leventopoulos G, Deftereos S (2014) Inflammation and chronic heart failure: from biomarkers to novel anti-inflammatory therapeutic strategies. Med Chem 10(7):682–699. 10.2174/1573406410666140318113325 25102199 10.2174/1573406410666140318113325

[87] Hanna P, Buch E, Stavrakis S et al (2021) Neuroscientific therapies for atrial fibrillation. Cardiovasc Res 117(7):1732–1745. 10.1093/cvr/cvab172 33989382 10.1093/cvr/cvab172 PMC8208752

[88] Margulescu AD, Mont L (2017) Persistent atrial fibrillation vs paroxysmal atrial fibrillation: differences in management. Expert Rev Cardiovasc Ther 15(8):601–618. 10.1080/14779072.2017.1355237 28724315 10.1080/14779072.2017.1355237

[89] Mohammed KS, Kowey PR, Musco S (2010) Adjuvant therapy for atrial fibrillation. Future Cardiol 6(1):67–81. 10.2217/fca.09.57 20014988 10.2217/fca.09.57

[90] Hulsmans M, Schloss MJ, Lee IH et al (2023) Recruited macrophages elicit atrial fibrillation. Science 381(6654):231–239. 10.1126/science.abq3061 37440641 10.1126/science.abq3061 PMC10448807

[91] Menzel A, Samouda H, Dohet F, Loap S, Ellulu MS, Bohn T (2021) Common and Novel Markers for Measuring Inflammation and Oxidative Stress Ex Vivo in Research and clinical practice-which to use regarding Disease outcomes? Antioxid (Basel) 10(3):414. 10.3390/antiox10030414 10.3390/antiox10030414 PMC8001241 33803155

[92] Matsumori A (2022) Targeting inflammation in the diagnosis, management, and Prevention of Cardiovascular diseases. Glob Heart 17(1):80. 10.5334/gh.1156 36382160 10.5334/gh.1156 PMC9635324

[93] Andrade J, Khairy P, Dobrev D, Nattel S (2014) The clinical profile and pathophysiology of atrial fibrillation: relationships among clinical features, epidemiology, and mechanisms. Circ Res 114(9):1453–1468. 10.1161/CIRCRESAHA.114.303211 24763464 10.1161/CIRCRESAHA.114.303211

[94] Huang M, Huiskes FG, de Groot NMS, Brundel BJJM (2024) The role of Immune cells driving Electropathology and Atrial Fibrillation. Cells 13(4):311. 10.3390/cells13040311 38391924 10.3390/cells13040311 PMC10886649

[95] Brandes A, Smit MD, Nguyen BO, Rienstra M, Van Gelder IC (2018) Risk factor management in Atrial Fibrillation. Arrhythm Electrophysiol Rev 7(2):118–127. 10.15420/aer.2018.18.2 29967684 10.15420/aer.2018.18.2 PMC6020195

[96] Méndez Hernández R, Ramasco Rueda F (2023) Biomarkers as prognostic predictors and therapeutic guide in critically ill patients: clinical evidence. J Pers Med 13(2):333. 10.3390/jpm13020333 36836567 10.3390/jpm13020333 PMC9965041

[97] Rani S, Dhar SB, Khajuria A et al (2023) Advanced overview of biomarkers and techniques for early diagnosis of Alzheimer’s Disease. Cell Mol Neurobiol 43(6):2491–2523. 10.1007/s10571-023-01330-y 36847930 10.1007/s10571-023-01330-y PMC11410160

[98] Rifai N, Ridker PM (2002) Inflammatory markers and coronary heart disease. Curr Opin Lipidol 13:383–389 12151853 10.1097/00041433-200208000-00005

