# Supplementary material for: Beyond the beats: a systematic review of the underlying inflammatory pathways between atrial fibrillation and cognitive decline
Source: Neurol Sci. 2025 Feb 20;46(7):2951–63. doi: 10.1007/s10072-025-08040-x (PMC12152057; doi:10.1007/s10072-025-08040-x)
Supplement: Supplementary file 1 — Supplementary Material 1 [file 10072_2025_8040_MOESM1_ESM.pdf]

Supplemental material.

Keywords used for the search.
Keywords were derived from the research literature. The search strategy combines the following terms:

1.PubMed

Filters: English/Spanish/Humans/From inception to 2024
Advanced search

(“atrial fibrillation*” OR “atrial flutter” OR “Afib” OR “Auricular Fibrillation*” OR “Heart Atrium Arrhythmia”)

AND (Cogn* OR “cognitive impairment*” OR dementia OR Alzheimer* OR “lewy bod*” OR “memory disorder*” OR “cognitive dysfunction*” OR “vascular dementia*” OR “frontotemporal lobar degenerat*”)

AND (“Inflammatory Process*” OR “Infec* Process*” OR “Mechanistic link*” OR “Inflammatory biomarker*” OR Inflammation* OR “C-reactive protein” OR PCR OR “Neutrophil Lymphocyte Ratio”)

2.Web of Science

Filters: English/Spanish/Article/From inception to 2024
Basic search: [Web of Science Core Collection]
Note. It does not allow to put Human as filter.

(“atrial fibrillation*” OR “atrial flutter” OR “Afib” OR “Auricular Fibrillation*” OR “Heart Atrium Arrhythmia”)

AND (Cogn* OR “cognitive impairment*” OR dementia OR Alzheimer* OR “lewy bod*” OR “memory disorder*” OR “cognitive dysfunction*” OR “vascular dementia*” OR “frontotemporal lobar degenerat*”)

AND (“Inflammatory Process*” OR “Infec* Process*” OR “Mechanistic link*” OR “Inflammatory biomarker*” OR Inflammation* OR “C-reactive protein” OR PCR OR “Neutrophil Lymphocyte Ratio”)

3.PsycINFO

Filters: Peer-reviewed/English/Spanish/ Humans/From inception to 2024
Advanced search

(“atrial fibrillation*” OR “atrial flutter” OR “Afib” OR “Auricular Fibrillation*” OR “Heart Atrium Arrhythmia”)

AND (Cogn* OR “cognitive impairment*” OR dementia OR Alzheimer* OR “lewy bod*” OR “memory disorder*” OR “cognitive dysfunction*” OR “vascular dementia*” OR “frontotemporal lobar degenerat*”)

AND (“Inflammatory Process*” OR “Infec* Process*” OR “Mechanistic link*” OR “Inflammatory biomarker*” OR Inflammation* OR “C-reactive protein” OR PCR OR “Neutrophil Lymphocyte Ratio”)
